# Current state of the opportunities for derivation of germ-like cells from pluripotent stem cells: are you a man, or a mouse?

**DOI:** 10.1080/13102818.2014.907037

**Published:** 2014-07-18

**Authors:** Rumena Petkova, Borislav Arabadjiev, Stoyan Chakarov, Roumen Pankov

**Affiliations:** ^a^ Scientific Technological Service (STS) Ltd., Sofia, Bulgaria; ^b^ Department of Cell Biology, Histology and Embryology, and Department of Biochemistry, Faculty of Biology, Sofia University ‘St. Kliment Ohridsky’, Sofia, Bulgaria

**Keywords:** mESC, hESC, pluripotency, ground state, PGC, germ cells

## Abstract

The concept of pluripotency as a prerogative of cells of early mammal embryos and cultured embryonic stem cells (ESC) has been invalidated with the advent of induced pluripotent stem cells. Later, it became clear that the ability to generate all cell types of the adult organism is also a questionable aspect of pluripotency, as there are cell types, such as germ cells, which are difficult to produce from pluripotent stem cells. Recently it has been proposed that there are at least two different states of pluripotency; namely, the naïve, or ground state, and the primed state, which may differ radically in terms of timeline of existence, signalling mechanisms, cell properties, capacity for differentiation into different cell types, etc. Germ-like male and female rodent cells have been successfully produced *in vitro* from ESC and induced pluripotent stem cells. The attempts to derive primate primordial germ cells (PGC) and germ cells *in vitro* from pluripotent stem cells, however, still have a low success rate, especially with the female germline. The paper reviews the properties of rodent and primate ESC with regard to their capacity for differentiation *in vitro* to germ-like cells, outlining the possible caveats to derivation of PGC and germ cells from primate and human pluripotent cells.

## The rise and fall of the concept of ‘tabula rasa’ for pluripotent stem cells

Let us then suppose the mind to be, as we say, white paper void of all characters.…How comes it to be furnished? … In one word, from experience.

John Locke, An Essay Concerning Human Understanding (1690)

Ever since it was first defined, pluripotency as a cellular state was believed to be quite straightforward in terms of its properties as well as its timeline – namely, a natural state typical of early embryonic cells, enabling them to generate all types of cells which make up the adult organism.[1,2] Later on it became clear that pluripotency could be induced in cells well past the natural stage of hyperplasticity typical of the cells of the early embryo and that terminally differentiated cells could easily be reprogrammed to pluripotency with only a limited set of protein factors and small molecules.[3–5] Further, it was demonstrated that the umbrella term of ‘pluripotency’ may actually cover very different types of cells, which share the ability to generate cell types characteristic of all three germ layers and to form teratomas when implanted in immunocompromised hosts, but may vary drastically in their expression profile, genome stability, capacity for self-renewal, propensity for differentiation, etc.[6–8] Finally, it was proposed that at least some types of embryonic stem cells (namely, rodent ESC) may exist in two alternative states – naïve (ground) and primed (for differentiation) pluripotent cells, which are different not only with regards to the time of extraction of the starting cellular material from early embryos but also in other essential properties such as the capacity to produce some types of cells, chromatin architecture, basic mechanisms of cellular signalling, the ability for integration into heterologous environments and for creating chimaeric blastocysts.[9,10] More specifically, it was proposed that rodent embryonic cells could be ‘naïve’, procured from pre-implantation blastocysts and ‘primed’, from the post-implantation epiblast (though pre-implantation blastocysts could also be used as a source of primed ESC), while primate ESC (derived from early blastocysts) had only one state, corresponding to the primed state, but with some shared characteristics typical of the naïve state, mainly in terms of cell signalling pathways.[9,11–13] The properties of naïve and primed pluripotent cells for differentiation into various cell types were found to be drastically different with regard to their capacity to differentiate to certain specialized cell types, such as germ cell precursors and true germ cells.[9,14]

The concept of existence of naïve and primed states is a source of inherent dualism, in the combined sense of comparative mammal physiology, basic cellular processes at molecular level as well as potential applications. At least in theory, the notion of existence of more than one type of pluripotent state may effectively resolve the long-standing mystery of the differences in the requirements for *in vitro* maintenance of the pluripotent state in ESC of related (albeit different) species such as rodents and primates (including humans). At the same time, however, it is likely to cancel out many hopes for direct translation of results achieved in murine and rat models to humans, as it has happened several times already (if we could only remember the infamous phenylbutylnitrone trial of 2005).[15,16] The question, or rather, the list of questions that logically derive from the concept of existence of two states of ESC is whether different species are really that different from each other biologically or might it be the simple fact that in a common mechanism there might be yet undiscovered components of the signalling pathways that are different and/or work in a different manner. As of now, the latter seems more likely than the former, though, considering some unique biological characteristics of rodents (e.g. diapause, ‘the rodent repairadox’, etc.), it is possible that at least some species may have their own special properties with regard to basic cell processes. There have been reports about isolation of pluripotent stem cells from species different from rodents, which exhibit properties characteristic of the ground state, but these are generally induced pluripotent stem cells, resulting from reprogramming of different cell types. Attempts to isolate ground state ESC from species other than mice and rats have been unsuccessful so far, preserving the typical naïve pluripotent ESC as a prerogative of rodents (see below).

## Basic differences between the naïve and the primed state in pluripotent cells

There's a divinity that shapes our ends,

Rough-hew them how we will.

William Shakespeare, Hamlet, Prince of Denmark (circa 1605), Act IV, scene 2.

Since the ground state as an alternative to the primed state has only been described in rodent ESC so far, the current body of research data is heavily biased towards mouse and rat models. It is still unclear whether ESC from other species may exhibit the same properties as rodent ESC, or whether the interspecies barrier is holding steady. In either case, sets of properties similar between naïve and primed ESC and which are clearly different between the naïve and the primed state of rodent ESC and, respectively, between rodent and primate ESC, have been compiled. Properties representative of the former group are, for example, the capacity to form teratomas when injected into immunocompromised hosts and the expression of the basic factors of pluripotency (Oct3/4, Sox2, Nanog).[17,18] Among the properties which are different between the ground- and the primed-state prominent are the activation status of the X chromosomes of female embryos (in naïve rodent ESC, both X chromosomes are active, whereas in primed rodent ESC and in primate ESC one X chromosome is promptly inactivated); the expression of some of the factors of the undifferentiated state; the ability to colonize host blastocysts thereby creating chimaeras (an essential functional characteristics of the ground state); response to basic signalling molecules, such as Lif/Stat3 (supporting self-renewal in naïve rodent ESC while having no effect in primed rodent ESC and primate ESC) and the Fgf/Erk (routing naïve rodent ESC towards differentiation; whereas, primed rodent ESC and primate ESC typically respond to fibroblast growth factor (FGF) with self-renewal), etc.[9,12,14,19]

## Scripta manent: peculiarities in the transition between the ground and the primed state

Men's courses will foreshadow certain ends … .

But if the courses be departed from, the ends will change.

Charles Dickens, A Christmas Carol (1843)

It has been speculated that ESC priming occurs in the early embryo in rough coincidence with the random inactivation of the X chromosome.[20–22] Indeed, in rodent naïve ESC, both X chromosomes of female embryos are active, and the inactivation of one of them signifies the transition to the primed state. Since the development of the blastocyst follows a very different pattern between rodents and other mammals, especially primates (e.g. formation of egg cylinder vs. formation of embryonic disk), there might be topological as well as temporal restrictions to the phase of ‘naïveté’, in other words naïve primate ESC may exist only as rare subpopulations and/or the moment when the naïve state exists may only be ephemeral and, hence, very difficult to capture. Human female ESC with two active X chromosomes have been maintained in conditions of relative hypoxia (5% oxygen content) and FGF supplementation, while control hESC grown in conditions of atmospheric oxygen content showed promptly inactivated X chromosome.[23] Attempts to derive naïve primate and hESC have not been successful so far, though it has been reported that a ‘naïve’ compartment characterized by two active X chromosomes has been identified in human ESC derived from female embryos deemed unusable for reproductive purposes after preimplantation genetic diagnosis (PGD).[23–25] This may mean that a transient naïve population exists, albeit transiently, in pre-implantation primate embryos. The FGF/extracellular signal-regulated kinases (ERK) as well as the dual-inhibitor (2i, or ERK: glycogen synthase kinase) cultural conditions, however, fails to suppress hypoblast specification in human embryos unusable because of genetic disease under conditions of physiological oxygen content as well as reduced oxygen content.[22,25] Notably, it has recently been reported that parthenote-derived ESC have been shown to exhibit variance in the pattern of X inactivation, with a distinct population that does not contain inactivated X chromosome.[26] Since the number of newly created xeno-free hESC lines is growing worldwide,[27–30] it could be expected that the definite proof of the existence of the naïve state of human ESC and the investigation of its specificities, conditions of *in vitro* maintenance and its properties is a question of the near future.

## Inconsistency of available data for different species

Data! Data! Data! (Sherlock Holmes) cried impatiently.

I can't make bricks without clay.

Arthur Conan Doyle, The Adventure of the Copper Beeches (1892)

Mice and rats are undoubtedly the most widely used (and, therefore, the best-studied) animal models; while primates (specifically, humans) are the most intensively researched object in modern biomedical science and in stem cell research. As of now, rodent studies provide most of the insight about how pluripotent stem cells work and what targeted effort is required to commit them *in vitro* to one cell lineage or another. Primate and human ESC are also studied extensively, though the progress in this field is often slowed down for ethical reasons. Derivation of ESC and establishment of ESC lines from animal species other than mice, rats and humans – primarily from domestic animals and lower primates – have been a long-standing goal, so far with varying results. As the domestic pig has been a long-standing model for research intended to be translated on humans, with its basic morphology, physiology and metabolism being close to humans’ and with its tissues tending to be the most compatible to human tissue, porcine ESC have been derived, but stable lines have not been established yet,[31,32] so that the focus of attention is recently shifting predominantly towards induced porcine ESC.[33,34] Equine ESC have been extracted back in 2002, which was driven mainly by the desire to create means for cell regeneration in joint and tendon injury in working and racing horses.[35–37] In 2009, isolation of primary bovine ESC has been reported.[38] Also, ESC have been derived from many other species, such as chicken, cattle and sheep.[39–41] There is a common issue, however, with the maintenance of all these ESC cultures and lines, which resides mainly in the want of information about the markers characterizing the undifferentiated state and the mechanisms responsible for its maintenance. Protocols for maintenance of ESC of species different from the mouse and the rat are generally still under development and, therefore, often prone to failure.[42,43] There has been considerable effort targeted at studying the properties of ESC from nonhuman primates, such as the cynomolgus monkeys and rhesus monkeys.[44–46]

There are several important aspects that very likely contribute to the difference in the success rate with rodent and primate pluripotent cells. For example, there are numerous genes which are critically important in human germline development, for which mice homologues do not exist – among these, for example, is the human Y chromosome gene *DAZ*. Also, despite the remarkable similarity between mammalian genomes (for example, mouse and human genomes have 70%–90% overall similarity,[47] the divergence between some of the important genes which play a role in germline development may be as low as 30%, as is the case with the *STELLA* gene.[48] Existing studies of the human genes involved in germline development which have their rodent homologues (e.g. *Dazla* mouse homologue for the *DAZL* gene in humans, *Boll* for *BOULE*, etc.) do not seem to be sufficient at the moment in order to reveal the reason for the observed differences in early germline development.[49,50]

All in all, the research data available at the moment is generally relevant for mice and rats, and, to a lesser extent, to humans. Until this huge gap in research data is filled at least partially, so that properties of pluripotent cells from animals other than rodents are studied and the conditions for their maintenance in the undifferentiated state are established and validated, there is not much hope for productive transfer of knowledge between species.

## Towards derivation of primordial germ cells and gametes from pluripotent stem cells

Nature has found only one method of organizing living matter.

There is, however, another method…which has not yet occurred to Nature at all.

Karel Čapek, RUR (1920)

If pluripotency is the ability to generate all types of cells which make up for the adult organism, ability to produce germ cells, in other words, haploid cells that could together create an embryo without use of cloning techniques could be viewed as the essence of pluripotency itself. Ability to create primordial germ cells (PGC) and gametes from undifferentiated cells would be an invaluable tool in studying the molecular mechanisms of infertility. It could be speculated that in the future, coupled with the techniques for induction of pluripotency in somatic cells which have been developed, this could provide an option for creating a gamete reserve in cases where the natural means for creating progeny have been damaged or destroyed (e.g. after cytotoxic therapies in individuals whose reproductive plans are unfinished or have not been considered, such as children and young adults) or, possibly, in cases where all routine options for assisted reproduction have failed. Naturally, the development and potentially the use of germ cells produced *in vitro* could be expected to produce significant ethical issues, and would be subject to very strict regulations, but since about half of the causes of infertility are considered to be linked to defects in germ cell production, it could be expected that the effort invested in the field of reproductive biomedicine of stem cells would be serious.

Differentiation of pluripotent cells to germ cells, however, presents a challenge to molecular and cell biology so far, though successes have been noted in the field already. Basically, there are two general approaches to generate germ-like cells and gametes; namely, the chimaera technology and the *in vitro* differentiation of undifferentiated cells – embryonic or induced pluripotent cells. Both approaches have their restrictions and both seem to be successful in rodent models and problematic in primates.

## Chimaera studies

What a chimera then is man! What a novelty!

Blaise Pascal, Pensées (1690)

Basically, the chimaera technique is relying on the chance that some of the ESC implanted in a host blastocyst would travel to the germinative ridges of the developing embryo, establishing the progenitor population of the germinative tissue of the resulting chimaeric organism. Murine and rat germ precursors and true germ cells, male and female alike have been successfully derived, mainly via creation of chimaeric animals, even rat–mouse chimeras.[51] The PGC have also been produced from ESC by co-culturing with embryonic gonad cells [52] or by reprogramming of primed ESC or even committed cells such as fibroblasts.[53,54] In other animal species, however, the attempts to produce chimaeric blastocysts by integration of ESC into a host embryo have been largely unsuccessful. Recently, Tachibana et al.[55] reported successful creation of chimaeric rhesus monkeys by an alternative method; namely, by aggregation of totipotent cells from different four-cell embryos. Even though creation of human chimaeras is strictly prohibited (at least under the current legislative framework) and, hence, experimental proof of principle is impossible, the ‘naïve vs. primed’ hypothesis presumes that primate and human ESC would a priori be incapable of integration in host blastocysts – at least, not with the conventional methods which readily apply to naïve rodent ESC.[56–59] Attempts to generate naïve primate and human stem cells are ongoing and, according to the reports, not with much success so far, as derivation of ‘true’ naïve ESC has only been reported for the mouse and the rat. There is still hope, nevertheless, that derivation of naïve ESC may be the solution to the current issue with creation of some specific types of cells from ESC (such as germinative cells) from a number of species, including primate ESC.

Much like primed rodent ESC, primate ESC are generally unable to colonize host blastocysts (or, at least, the attempts with nonhuman primates have been unsuccessful so far). Therefore, current chances of producing chimaeric primate embryos and germinative cells originating from the integrated primate ESC by the means routinely used in chimaera technique are very low. Blastocyst integration has been attempted with primed rodent ESC so far, the result being very low rate of integration with subsequent massive apoptosis of the foreign ESC.[60,61] Since primate ESC are closer to primed rodent ESC, hypothetically the same outcome could be expected, and experiments using rhesus monkeys as an animal model closer to humans than rats and mice did not produce different results.[55] Notably, co-culturing of primed mouse epiblast stem cells with gonad cells has been reported to result in generation of PGC precursors and true germ cells.[62]

## *In vitro* differentiation for creation of germ-like cells

I am the Fate's lieutenant: I act under orders.

Herman Melville, Moby Dick (1851)

The first reports of successful *in vitro* differentiation of mouse PGC from pluripotent cells appeared in 2003.[63,64] Again using mice as model system, in 2004 Geijsen et al.[65] produced male germ cells from murine ESC lines, which, when injected into mouse oocytes, produced viable blastocysts and live newborn mice – admittedly, with traits suggesting epigenetic deregulation of imprinting.[66] Ever since, male and female murine germ cells from pluripotent cells have been successfully derived and the respective culturing conditions and differentiation protocol established and validated.[67,68] ESC from nonhuman primates such as the cynomolgus monkey have been shown to be able to spontaneously differentiate into germ-like cells, albeit at a low rate,[69] or after culturing in nutrient medium conditioned by mouse gonad cells.[70,71]

At the present moment, the means for production of human germinative cells from pluripotent cells for research purposes lies in targeted *in vitro* differentiation of pluripotent cells to PGC and germ cells. Admittedly, the success rate with human cells is much lower than with rodent cells. First successful attempts to create human germ-like cells from undifferentiated cells date back to 2004, when Clark et al.[72] reported spontaneous differentiation of male and female cultured human ESC into pre-meiotic and, albeit in much lower percentage, postmeiotic germ cell precursors. Authors noted specifically that, unlike murine cells, human germ cells produced by differentiation from pluripotent cells seem to be expressing both the ‘male’ and the ‘female’ genetic programmes, regardless of their XX or XY karyotype. Putative PGC were selected for germline-specific markers such as DEAD/HBox4 (DDX4, VASA); Deleted in Azoospermia (DAZ), Deleted in Azoospermia-like (DAZL) and Boll-like (BOULE) or other molecules (e.g. epithelial cell adhesion molecule (EpCAM)), [73] and post-meiotic cells were identified by the presence of proteins characteristic of the synaptonemal complex – e.g. synaptonemal complex protein 3 (SYCP3), or other markers, such as PIWI-like 1 (PIWIL1).[74] The efficiency of spontaneous generation of PGC from pluripotent cells was found to be increased by induction of bone morphogenic protein/wingless type MMTV integration site family 3A (BMP/WNT3A) signalling pathway by BMP4, BMP7 and BMP8b, and subsequent selection for certain markers (e.g. octamer-binding transcription factor 4 (OCT4)/EpCam);[73] or by BMP supplementation/co-cultivation with fetal gonad cells,[50,75–78] though reportedly the percentage of human induced pluripotent stem cells (iPSC) that eventually differentiated into PGC using the above methods did not exceed 5%.[77] In 2009, Park et al.[79] produced female human PGC from hESC and induced pluripotent stem cells using modified culturing conditions with human fetal gonad stromal cells and noted that the erasure of the genetic imprint was initiated in PGC produced from hESC at day 7 of differentiation but not in PGC differentiated from iPSC. In 2011, Panula et al.[77] produced post-meiotic male and female human germ cell precursors from iPSC generated by dedifferentiation of fetal and adult somatic cells. These cells, however, expressed acrosin, a protein characteristic of spermatid acrosome, regardless of the XX or XY karyotype, corroborating the early findings of Clark et al.[72] Recently, it has been shown that under culturing conditions specific for spermatogonial stem cells (SSC conditions, initially developed and tested with murine ESC), human pluripotent stem cells differentiated into haploid, acrosin-positive cells expressing markers characteristic of round spermatids, though the yield is still admittedly in the order of several percent.[74] All in all, the success with producing human germ-like cells so far is significantly more advanced with male than with female germ cells, as the reports so far have been for generation of spermatogonia, spermatocytes and haploid round spermatids from pluripotent stem cells, while the generation of human female germ cells lags behind significantly. According to literature, protocols for differentiation of oocyte-like cells from pluripotent stem cells have seen the light, though the success is rather patchy and, once again, phenomenology largely governs the field.[80–82] All in all, despite the tremendous effort for creating germ cells from pluripotent cells, it still remains more of a question of chance than of skill.

## Conclusions

Derivation of germ-like cells from pluripotent cells is a goal that has been achieved with variable success for different types of pluripotent cells. Rodent PGC able to colonize sterilized gonads and true germ cells have been created successfully *in vivo* (using chimaera technology) as well as *in vitro*, via targeted differentiation and stringent selection. The methods for creation of pluripotent stem cell lines from animal species other than mice and rats (and of germ-like cells from animal pluripotent cells) are still under research and the success rate varies significantly. The relative success of derivation of different human cell types, including germ-like cells, is higher at the moment with human pluripotent cells other than ESC, which is likely to be related partly to fewer ethical issues related to the establishment and use of iPSC (no embryos destroyed; creation of gametes from somatic cells for the purposes of infertility treatments likely to be justified) and partly to methodological and technical difficulties related to peculiarities of the undifferentiated state in primate ESC. Apparently, the road to producing primate PGC and, later, gametes for research as well as applied purposes lies through differentiation of induced pluripotent cells rather than using embryonic sources. 
